# Antibiotic Resistance of Diverse Bacteria from Aquaculture in Borneo

**DOI:** 10.1155/2016/2164761

**Published:** 2016-09-25

**Authors:** M. M. Kathleen, L. Samuel, C. Felecia, E. L. Reagan, A. Kasing, M. Lesley, S. C. Toh

**Affiliations:** Department of Molecular Biology, Faculty of Resource Science and Technology, Universiti Malaysia Sarawak, 94300 Kota Samarahan, Sarawak, Malaysia

## Abstract

The administration of antimicrobials in aquaculture provides a selective pressure creating a reservoir of multiple resistant bacteria in the cultured fish and shrimps as well as the aquaculture environment. The objective of this study was to determine the extent of antibiotic resistance in aquaculture products and aquaculture's surrounding environment in Sarawak, Malaysian Borneo. Ninety-four identified bacterial isolates constituted of 17 genera were isolated from sediment, water, and cultured organisms (fish and shrimp) in selected aquaculture farms. These isolates were tested for their antibiotic resistance against 22 antibiotics from several groups using the disk diffusion method. The results show that the highest resistance was observed towards streptomycin (85%, *n* = 20), while the lowest resistance was towards gentamicin (1.1%, *n* = 90). The multiple antibiotic resistant (MAR) index of the isolates tested ranged between 0 and 0.63. It was suggested that isolates with MAR index > 0.2 were recovered from sources with high risk of antibiotic resistant contamination. This study revealed low level of antibiotic resistance in the aquaculture bacterial isolates except for streptomycin and ampicillin (>50% resistance, *n* = 94) which have been used in the aquaculture industry for several decades. Antibiotic resistant patterns should be continuously monitored to predict the emergence and widespread of MAR. Effective action is needed to keep the new resistance from further developing and spreading.

## 1. Introduction

Since the discovery of penicillin in 1928 by a Scottish scientist Alexander Fleming followed by the release of many other earlier drugs onto the market to treat infection, the development of drug resistance in various sectors including aquaculture has been reported [1–3]. The misuse and abuse of the antimicrobial drugs are among the important factors that have contributed to the rise of resistant microbes around the world. Antibiotics, which have saved millions of lives and were also known as miracle drug in the past, are no longer the ultimate way for the treatment of infections because bacteria have continued to develop multiple resistance towards many different types or classes of the drugs [4].

Antimicrobial agents have been widely used in fish farming for either therapeutic, prophylactic, or other purposes [5]. The antibiotics are normally used to increase growth as well as feed efficiency in the animals [6]. However, some of the antibiotics have been frequently used in both veterinary and human medicine such as sulfonamides, chloramphenicol, tetracycline, nitrofurans [5], oxytetracycline [7], neomycin, erythromycin, streptomycin, prefuran, and enrofloxacin [8]. The evolution of bacteria towards antibiotic resistance has been accelerated distinctly by selective pressure due to inappropriate and overuse of the antibiotics [3, 9]. In the efforts to cope with this problem, scientists have accelerated the search for alternative antimicrobial agents by screening many potential sources including medicinal plants [10, 11] and microbes [12, 13].

Aquaculture is an important sector in the agriculture industry and is rapidly growing to meet the world's demands for protein source. This sector is challenged with the diverse type of diseases and bacterial infections; and antibiotics are an excellent tool to circumvent the problem [14, 15]. The presence of bacteria with multiple antibiotic resistance found in food products has become a threat to public health as there is potential that the carried or acquired genes are transferred to other bacteria of clinical significance [16–18]. Some antibiotics which are commonly used in food-producing animals are also used in human medicine, reducing the antibiotic's efficiency when treating infections and increasing the morbidity and mortality associated with diseases. The resistance limits the choice of antibiotics for the disease treatment [4, 16, 19, 20].

The use of antibiotics needs to be monitored from time to time to evaluate the emergence and spread of bacterial resistance towards antimicrobial agents [5, 21]. There is limited data on the antibiotic resistance of bacteria in fish and other cultured organisms sampled directly from fish farms as well as the aquaculture environment. Therefore, this study aims to determine this.

## 2. Materials and Methods

### 2.1. Bacterial Strains

Sampling was carried out at aquaculture farms located at selected districts in Sarawak, Malaysian Borneo, including Kuching, Bintulu, Limbang, Miri, and Sampadi (Lundu). Three types of samples were collected which were the sediment, water, and cultured species. Cultured species refers to fish or shrimp. In Kuching, Bintulu, and Miri, the cultured organisms collected were the fish while in Limbang and Sampadi (Lundu), the cultured organisms collected were shrimps. Sampling and sample processing were performed according to standard operating protocol by [22]. The isolation, designation, and identification of the isolates were carried out as reported by Kathleen and coworkers earlier [23]. The list of bacteria and their source of origin are listed in Table 1.

### 2.2. Antibiotic Susceptibility Tests

Ninety-four bacterial isolates from 17 different genera [23] were assessed for their susceptibility to different antibiotics utilizing the disk diffusion method according to method described by Clinical and Laboratory Standards Institutes (CLSI) [24] on Mueller-Hinton agar (MHA). The bacterial groups and the antibiotics tested are listed in Table 1. Briefly, fresh bacterial culture with 0.5 McFarland turbidity was swabbed onto the MHA surface using sterile cotton buds. Commercial antimicrobial discs (Oxoid, UK) were evenly embedded onto the inoculated agar incubated at 37°C for 18 to 24 hours.* Escherichia coli* strain from American Type Culture Collection (ATCC) 25922 was used as control.

### 2.3. Data Collection and Analysis

The diameter of complete inhibition zone formed around the antibiotic discs was measured to the nearest whole millimeter using standardized ruler. The results obtained were analyzed as resistant or susceptible according to standard interpretative table by CLSI [25] and Bonnet [26]. Multiple antibiotic resistance (MAR) index was then determined for each isolate by dividing the number of antibiotics to which an isolate is resistant with the total number of antibiotics tested [2]. The MAR index is an indicator to identify the risk contamination that is potentially hazardous to human. Calculated value of more than 0.2 indicates that the isolates were isolated from high risk sources [27].

## 3. Results

In this study, commonly used antibiotics in both veterinary and human medicine were selected for the antibiotic susceptibility testing. The antibiotic selection also depends on the bacterial genera because different bacterial genera require different classes of antibiotics for optimal antibacterial activity. Some of the antibiotics were not tested in this study because some bacterial genera are naturally resistant to certain classes of antibiotics; hence the antibiotics were excluded from the analysis. The antibiotic profile of the bacterial isolates from the aquaculture fish and shrimps and their environment is shown in Table 1.

The bacterial isolates showing the top five highest percentages of resistant were towards streptomycin (85%, *n* = 20), followed by ampicillin (56.8%, *n* = 74), penicillin (47.1%, *n* = 51), erythromycin (43.1%, *n* = 51), and cephalotin (42.3%, *n* = 71). The bacterial isolates showing the top five highest percentages of susceptible were towards gentamicin (1.1%, *n* = 90), followed by tobramycin (2.2%, *n* = 90), chloramphenicol (4.0%, *n* = 75), norfloxacin (5%, *n* = 80), and amikacin (5.6%, *n* = 90). The other bacterial isolates and their percentage of resistant are shown in Table 1.

In this study, the antibiotic resistant patterns for all isolates were also determined to monitor the spread of antibiotic resistance. Sixty-one different antibiotic resistance patterns were observed among the isolates through this study (data not shown). The resistance patterns were highly variable; 20.2% (*n* = 94) isolates have no resistance towards any antibiotics tested, 16% (*n* = 94) isolates were resistant to only one antibiotic, and 63.8% (*n* = 94) isolates were resistant to multiple antibiotics.* Chryseobacterium* spp. isolated from water in Limbang aquaculture farm was resistant towards 12 out of 19 antibiotics tested, which was the highest amount of antibiotic resistance observed in this study.

Most isolates (53.2%, *n* = 94) possess distinctive pattern. However, there are also patterns (11 patterns) shared by 2 or more bacteria isolates. The pattern shared by most isolates (19 isolates, *n* = 94) is 0% resistance (MAR index equal to 0). There is at least an isolate with 0% resistance towards all antibiotics tested in all sampling locations except for Bintulu. There is difference in the resistance pattern for bacteria isolated from water, sediment, and the cultured organisms. Bacteria that were isolated from the same pond and same source of origin (sediment, water, or cultured organisms) possess different antibiotic patterns. This may be due to the genus and species difference of the bacteria isolates. The antibiotic resistant patterns of the bacterial genera are more influenced by the location from where the isolates were isolated rather than their genus.

**Figure 1:**
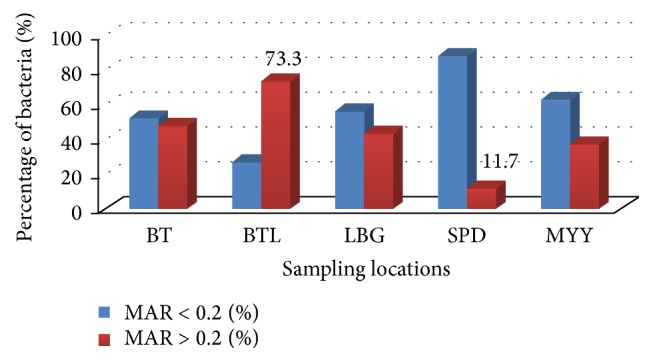
MAR index analysis based on sampling locations; BT: Batu Tujuh (Seven Miles), BTL: Bintulu, LBG: Limbang, SPD: Sampadi, and MYY: Miri.

Multiple antibiotic resistant (MAR) index analysis was introduced by Krumperman in 1983 [27]. This analysis has been used to group the different sources from where the bacteria were recovered using the frequency of antibiotics resistance [28]. Isolates with MAR < 0.2 were determined as isolates recovered from low risk sources of contamination while isolates with MAR > 0.2 were from high risk sources [27]. In this study, the MAR index ranged from 0 to 0.63. Analysis on overall isolates regardless of sampling location revealed that 63.1% (*n* = 94) isolates belong to group MAR < 0.2 while 36.9% (*n* = 94) isolates belong to group MAR > 0.2. MAR index was also assessed according to the sampling location (Figure 1). Bintulu (BTL) has the highest percentage (73.3%, *n* = 16) of bacteria isolated from high antibiotic-contaminated sources while Sampadi (SPD) has the lowest percentage (11.7%, *n* = 17).

## 4. Discussion

Assessment of the antibiotic resistance among aquaculture bacteria against antimicrobial agents is important for update on the bacterial antibiotic resistance patterns. It is part of a surveillance system aiming at monitoring emerging antibiotic resistant bacteria and their widespread. Isolation of antibiotic resistant bacteria from aquaculture products and aquaculture environment indicates the health risk associated with the aquaculture. There had been reports on detection of antibiotic resistance genes in bacteria isolated from aquaculture products that can be transferred to human microbiota. This matter is becoming critical if the resistance genes are transferrable to human pathogens. Providing effective treatment towards this infection becomes a problem to the medical practitioners as the choice of antibiotics is limited. Thus, any source of antibiotic resistant bacteria must be carefully monitored [4].

In antibiotic resistance analysis, the history of antibiotic application in particular area is reflected by the percentage of bacterial resistance to antibiotics [21]. The frequency of antibiotics usage is related to the level of resistance among bacteria [2, 29]. In this present study, high percentage of susceptibility was observed towards gentamicin, tobramycin, and chloramphenicol. Similarly, Lim and Kasing [21] and Hatha et al. [17] in their respective studies observed that almost none of the bacteria tested were resistant to gentamicin and chloramphenicol. Low frequency of antibiotic resistant bacteria may indicate the less activity associated with the contamination of antibiotics in the area.

The use of chloramphenicol in aquaculture has been banned in certain countries including Malaysia, Korea, and Japan since 1983 [30]. This is because of the adverse effect of chloramphenicol in humans, even at very low dosage, which can cause other side effects like severe or fatal blood problems. The problems associated with blood are like anemia and “grey syndrome,” a syndrome of cyanosis and cardiovascular collapse, which occurs particularly in newborn babies. This poses risks to the workers handling the products containing this antibiotic [5, 31]. Banning of antibiotics has aided in reducing the number of antibiotic resistant bacteria in an environment. This present study revealed very low percentage of resistance towards chloramphenicol (4%). Aarestrup et al. [32] reported the significant reduction in the frequency of vancomycin-resistant* Enterococci* from broiler after the banning of vancomycin in Denmark in 1995. This proves that government involvement in preventing dissemination of antibiotic resistant bacteria is important.

High percentage of streptomycin, ampicillin, and penicillin G resistance was observed in this study. Similarly, high ampicillin and streptomycin resistance were also observed by Zhang et al. [33] in their study on antibiotic resistance detection in* E. coli* strains isolated from two different aquaculture systems in South China. Hatha et al. [17] also recorded high resistance of ampicillin. In another study, identified isolates from mangrove soil in Malaysia were 100% resistant towards ampicillin and penicillin while 77.8% of the isolates were resistant towards streptomycin [34]. Son et al. [35] in their study on* Aeromonas hydrophila* isolated from* Tilapia mossambica *recorded 100% ampicillin resistance and 57% streptomycin resistance. Abdullahi et al. [36] observed 100% ampicillin resistance in* Pseudomonas* spp. isolated from Sarawak aquaculture environment. A study carried out by Akinbowale et al. [15] recorded 54.8% ampicillin resistance of aquaculture bacteria in Australia. In contrast with the results in this study, low resistance of streptomycin (21.2%) and ampicillin (6.1%) was observed in a study on 33 marine bacteria isolates by You et al. [37].

In this study, farmers in the aquaculture farms where the sampling was carried out stated that there was no history of utilization of antibiotics in their farms. Despite the absence of antibiotics as medicines or in feeds, high resistance was observed to commonly used antibiotics such as streptomycin, ampicillin, and penicillin. High resistance of ampicillin and streptomycin in this study and other researches was not surprising as these antibiotics were among the first antibiotics introduced since the discovery of penicillin [38]. Although antibiotic usage in the studied farms has been stopped decades ago, antibiotic contamination is still possible as there may still be residues of antibiotics left in the environment. Bacteria isolated from the sediment of the aquaculture pond may have acquired antibiotic resistance characteristics through unconsumed foods and the cultured organism's faeces that contain the remaining antibiotics [39–41]. The unconsumed foods and faeces will be deposited in the sediment and the composition of sediment microbiota will be altered due to selective pressure [39]. Fish feeds were a possible reservoir for antibiotic resistant genes in the farm sediments [41].

In this present study, Bintulu (BTL) recorded the highest percentage (73.3%, *n* = 16) of bacteria isolated from high antibiotic-contaminated sources (MAR > 0.2) while Sampadi (SPD) aquaculture farm recorded the least number (11.7%, *n* = 17) of MAR index > 0.2 isolates. The high number of bacteria with MAR > 0.2 was found in Bintulu. The aquaculture farm is located at an area that has many agriculture activities (e.g., pig farming, duck farming, and dragon fruit cultivation) surrounding it. There is the possibility that antibiotics from the animal feeds or medications were absorbed into the sediment causing bacterial selection in the nearby environment. Multiple antibiotic resistant bacteria might have travelled through water from these agriculture activities to the aquaculture ponds. Buschmann et al. [42] in their study suggested that antibiotic resistant bacteria in mariculture farm may be transported by water current which flows from surrounding farms that utilize antibiotics excessively. Antibiotic resistance pattern may vary depending on the geographical locations and selective pressure [43, 44] and these patterns change rapidly from time to time.

The different patterns exhibited by different strains or species suggest how complex is the understanding of the antibiotics resistance in the study area. In this study, the resistance patterns were highly variable; 20.2% (*n* = 94) isolates have no resistance towards any antibiotics tested, 16% (*n* = 94) isolates were resistant to only one antibiotic, and 63.8% (*n* = 94) isolates were resistant to multiple antibiotics.

Awareness on antibiotic resistance threat should be instilled in the community regardless of age as precaution and prevention step against dissemination of antibiotic resistant bacteria. The community must be educated on antibiotics and their effects on public health. Many surveillance programs had also been introduced to monitor the emergence and spread of antibiotic resistant bacteria. It has also been suggested by Son et al. [4] that plasmid screening should be considered as an additional procedure in the monitoring programs to trace antibiotic resistance dissemination. Alternatives to treatment using antibiotics like probiotics, vaccines, and antimicrobials from plants should be also considered. However, most of the alternatives could not really effectively replace antibiotics, so they act as additional preventive measures rather than alternatives.

## 5. Conclusion

The MAR indexing has revealed that 63.1% of the isolates came from low antibiotic usage area. Although antibiotic resistance in aquaculture in the Malaysian Borneo is still in its infancy, the need for continuous monitoring of the antibiotic resistance patterns should not be overlooked and the community should be educated on the awareness of antibiotic resistance and its implication on human health and environment.

## Figures and Tables

**Table 1:** Antibiotic profile and MAR index of isolates from aquaculture.

Isolates code and species		Antibiotics																						MAR index
		Ak	Tob	CN	K	S	Te	DO	P	PRL	AMP	CIP	NOR	CRO	CAZ	KF	IPM	SXT	RD	NA	C	F	E	
BT-F14	*Edwardsiella tarda *	−	−	−	−	+	−	−	nt	+	+	−	−	−	−	+	+	−	nt	−	−	+	nt	0.32
BTL-2W3	*Enterobacter* sp.	−	−	−	+	+	−	−	nt	−	+	−	−	−	−	+	+	−	nt	+	−	+	nt	0.37
BTL-2W4	*Enterobacter* sp.	+	−	−	+	+	−	−	nt	−	+	+	−	−	−	+	+	−	nt	+	−	+	nt	0.47
MYY-W9	*Enterobacter hormaechei *	−	−	−	+	+	−	−	nt	−	+	−	−	−	−	+	−	−	nt	−	−	+	nt	0.26
LBG-2P6	*Serratia liquefaciens *	−	−	−	−	+	−	−	nt	+	+	−	−	−	−	+	−	−	nt	−	−	+	nt	0.26
LBG-2W5	*Serratia marcescens *	−	−	−	−	+	+	+	nt	−	+	+	−	−	−	+	+	−	nt	−	−	+	nt	0.42
MYY-F9	*Escherichia coli *	−	−	−	+	+	−	−	nt	−	+	−	−	−	−	−	−	−	nt	−	−	−	nt	0.16
BT-F7	*Escherichia albertii *	−	−	−	−	+	+	+	nt	+	+	+	−	−	−	+	−	+	nt	+	−	−	nt	0.47
BTL-W8	*Klebsiella pneumoniae *	−	−	−	+	+	−	−	nt	+	+	+	−	−	−	+	+	−	nt	+	−	+	nt	0.47
BT-2W2	*Klebsiella pneumoniae *	−	−	−	−	+	+	+	nt	+	+	+	−	−	−	+	−	−	nt	−	−	+	nt	0.42
BT-2S3	*Plesiomonas shigelloides *	−	−	−	−	+	−	−	nt	−	−	+	+	−	−	−	−	−	nt	+	−	+	nt	0.26
BT-F1	*Plesiomonas shigelloides *	−	−	−	−	−	−	−	nt	−	−	+	−	+	+	−	−	−	nt	+	−	−	nt	0.21
BT-F6	*Plesiomonas shigelloides *	−	−	−	−	+	−	−	nt	−	+	−	−	−	−	+	+	−	nt	−	−	+	nt	0.26
BT-F10	*Plesiomonas shigelloides *	−	−	−	−	−	−	−	nt	−	+	+	−	−	−	+	−	−	nt	−	−	+	nt	0.21
BT-F13	*Plesiomonas shigelloides *	−	−	−	−	+	−	−	nt	−	+	−	−	−	−	+	+	−	nt	−	−	+	nt	0.26
BTL-F4	*Plesiomonas shigelloides *	+	−	−	+	+	−	−	nt	+	+	−	−	−	−	−	−	−	nt	−	−	−	nt	0.26
BTL-F10	*Plesiomonas shigelloides *	−	−	−	−	−	−	−	nt	+	+	+	−	+	+	−	−	−	nt	−	−	−	nt	0.26
BTL-2F4	*Plesiomonas shigelloides *	−	−	−	−	+	−	−	nt		+	+	−	−	−	+	+	−	nt	−	−	+	nt	0.32
BTL-2F7	*Plesiomonas shigelloides *	−	−	−	−	+	−	−	nt	−	+	−	−	−	−	+	+	−	nt	−	−	+	nt	0.26
BT-2F10	*Pseudomonas putida *	−	−	−	nt	nt	nt	nt	nt	−	nt	−	−	nt	−	nt	−	nt	+	nt	nt	nt	nt	0.11
BT-2F5	*Pseudomonas putida *	−	−	−	nt	nt	nt	nt	nt	−	nt	−	−	nt	−	nt	−	nt	−	nt	nt	nt	nt	0.00
MYY-S5	*Pseudomonas* sp.	−	−	−	nt	nt	nt	nt	nt	−	nt	−	−	nt	−	nt	−	nt	+	nt	nt	nt	nt	0.11
BT-2S9	*Aeromonas hydrophila *	−	−	−	nt	nt	nt	nt	nt	+	nt		+	nt	+	nt	−	nt	−	nt	nt	nt	nt	0.33
BT-F12	*Aeromonas hydrophila *	−	−	−	nt	nt	nt	nt	nt	−	nt	−	+	nt	−	nt	−	nt	−	nt	nt	nt	nt	0.11
BT-F15	*Aeromonas jandaei *	−	−	−	nt	nt	nt	nt	nt	−	nt	−	−	nt	+	nt	−	nt	−	nt	nt	nt	nt	0.11
MYY-2F8	*Aeromonas jandaei *	−	−	−	nt	nt	nt	nt	nt	+	nt	+	−	nt	+	nt	−	nt	+	nt	nt	nt	nt	0.44
MYY-2F4	*Aeromonas punctata *	−	−	−	nt	nt	nt	nt	nt	−	nt	−	−	nt	−	nt	−	nt	+	nt	nt	nt	nt	0.11
MYY-2W3	*Aeromonas jandaei *	−	+	−	nt	nt	nt	nt	nt	−	nt	−	−	nt	−	nt	+	nt	−	nt	nt	nt	nt	0.22
LBG-W6	*Stenotrophomonas maltophilia *	nt	nt	nt	nt	nt	−	nt	nt	nt	nt	−	nt	nt	nt	nt	+	−	+	nt	+	nt	nt	0.60
LBG-2W2	*Chryseobacterium* sp.	+	+	+	+	+	−	−	nt	+	+	−	−	+	+	+	+	−	nt	−	−	+	nt	0.63
SPD-P7	*Vibrio rotiferianus *	nt	nt	nt	nt	nt	−	nt	nt	nt	−	nt	nt	nt	nt	nt	nt	−	nt	nt	−	nt	nt	0.00
SPD-S6	*Vibrio fischeri *	nt	nt	nt	nt	nt	−	nt	nt	nt	−	nt	nt	nt	nt	nt	nt	−	nt	nt	−	nt	nt	0.00
SPD-W5	*Vibrio* sp.	nt	nt	nt	nt	nt	−	nt	nt	nt	−	nt	nt	nt	nt	nt	nt	−	nt	nt	−	nt	nt	0.00
BT-W2	*Acinetobacter baumannii *	+	−	−	nt	nt	+	−	nt	+	nt	−	nt	+	−	nt	−	+	nt	nt	nt	nt	nt	0.45
BT-2W8	*Acinetobacter* sp.	−	−	−	nt	nt	−	−	nt	−	nt	−	nt	−	−	nt	−	−	nt	nt	nt	nt	nt	0.06
BT-2W9	*Acinetobacter baumannii *	−	−	−	nt	nt	−	−	nt	−	nt	−	nt	−	+	nt	−	−	nt	nt	nt	nt	nt	0.09
BTL-2W2	*Acinetobacter baumannii *	+	−	−	nt	nt	−	−	nt	+	nt	−	nt	+	+	nt	−	−	nt	nt	nt	nt	nt	0.36
BT-2F9	*Acinetobacter calcoaceticus *	−	−	−	nt	nt	−	−	nt	+	nt	−	nt	+	+	nt	−	−	nt	nt	nt	nt	nt	0.27
BTL-2F1	*Acinetobacter* sp.	−	−	−	nt	nt	−	−	nt	+	nt	−	nt	+	−	nt	−	−	nt	nt	nt	nt	nt	0.18
MYY-2W4	*Acinetobacter* sp.	−	−	−	nt	nt	−	−	nt	+	nt	−	nt	+	−	nt	−	−	nt	nt	nt	nt	nt	0.18
LBG-2W3	*Acinetobacter* sp.	−	−	−	nt	nt	−	−	nt	−	nt	−	nt	−	−	nt	−	−	nt	nt	nt	nt	nt	0.00
SPD-2P1	*Acinetobacter* sp.	−	−	−	nt	nt	−	−	nt	−	nt	−	nt	+	−	nt	−	−	nt	nt	nt	nt	nt	0.09
SPD-2P8	*Acinetobacter calcoaceticus *	−	−	−	nt	nt	−	−	nt	−	nt	−	nt	−	−	nt	−	−	nt	nt	nt	nt	nt	0.00
BTL-F1	*Staphylococcus* sp.	−	−	−	−	nt	−	nt	+	nt	+	−	−	+	+	−	−	−	−	nt	−	−	+	0.26
MYY-W6	*Staphylococcus sciuri *	−	−	−	−	nt	−	nt	−	nt	+	−	−	+	+	−	−	−	−	nt	−	−	−	0.16
MYY-2F2	*Staphylococcus xylosus *	−	−	−	−	nt	−	nt	+	nt	+	−	−	−	−	+	+	−	+	nt	−	−	+	0.32
LBG-P2	*Staphylococcus* sp.	−	−	−	−	nt	−	nt	−	nt	−	−	−	−	−	−	−	−	−	nt	−	−	+	0.05
LBG-2P3	*Staphylococcus saprophyticus *	−	−	−	−	nt	−	nt	+	nt	+	−	−	+	+	−	−	+	−	nt	−	−	+	0.32
LBG-2P7	*Staphylococcus xylosus *	−	−	−	−	nt	−	nt	+	nt	+	−	−	+	+	+	−	−	−	nt	−	−	+	0.32
SPD-W7	*Staphylococcus* sp.	−	−	−	−	nt	−	nt	−	nt	−	−	−	−	−	−	−	−	−	nt	−	−	+	0.05
SPD-W3	*Staphylococcus* sp.	−	−	−	−	nt	−	nt	+	nt	−	−	−	−	−	−	−	−	−	nt	−	−	+	0.11
SPD-W1	*Staphylococcus saprophyticus *	−	−	−	−	nt	−	nt	+	nt	+	−	−	+	+	−	−	−	−	nt	−	−	−	0.21
SPD-P1	*Staphylococcus* sp.	−	−	−	−	nt	−	nt	+	nt	−	−	−	+	+	−	−	−	−	nt	−	−	−	0.16
SPD-S9	*Staphylococcus saprophyticus *	−	−	−	−	nt	−	nt	−	nt	−	−	−	−	−	−	−	−	+	nt	−	−	−	0.05
BT-S1	*Bacillus* sp.	−	−	−	−	nt	−	−	−	nt	−	−	−	−	−	−	−	−	−	nt	−	−	−	0.00
BT-S2	*Bacillus* sp.	−	−	−	−	nt	+	+	+	nt	+	−	−	−	−	+	−	−	+	nt	−	+	+	0.42
BT-S3	*Bacillus* sp.	−	−	−	−	nt	−	−	−	nt	−	−	−	−	−	−	−	−	−	nt	−	−	−	0.00
BT-S4	*Bacillus* sp.	−	−	−	−	nt	−	−	−	nt	−	−	−	−	−	−	−	−	−	nt	−	−	−	0.00
BT-2S1	*Bacillus cereus *	−	−	−	−	nt	−	−	+	nt	+	−	−	+	+	+	−	+	+	nt	−	+	−	0.42
BT-2S5	*Bacillus megaterium *	−	−	−	−	nt	−	−	−	nt	+	−	−	−	−	−	−	−	+	nt	−	−	−	0.11
BT-2S8	*Bacillus megaterium *	−	−	−	−	nt	−	−	−	nt	−	−	−	−	+	−	−	−	−	nt	−	−	+	0.11
BT-2S10	*Bacillus aryabhattai *	−	−	−	−	nt	−	−	−	nt	+	−	−	−	−	−	−	−	−	nt	−	−	−	0.05
BTL-S1	*Bacillus* sp.	−	−	−	−	nt	−	−	+	nt	+	−	−	+	+	+	−	+	+	nt	−	−	−	0.37
BTL-S3	*Bacillus pumilus *	−	−	−	−	nt	−	−	+	nt	+	−	−	+	+	−	−	−	+	nt	+	−	+	0.37
BTL-2S4	*Bacillus* sp.	−	−	−	−	nt	−	−	+	nt	+	−	−	−	−	+	−	−	+	nt	−	−	+	0.26
BTL-2S8	*Bacillus* sp.	−	−	−	−	nt	−	−	+	nt	+	−	−	+	+	−	−	−	−	nt	−	−	−	0.21
BTL-2S9	*Bacillus pumilus *	−	−	−	−	nt	−	−	−	nt	−	−	−	−	+	−	−	−	−	nt	−	+	−	0.11
BT-2W5	*Bacillus megaterium *	−	−	−	−	nt	−	+	+	nt	+	−	−	−	−	+	−	−	+	nt	−	+	+	0.37
BTL-2W7	*Bacillus* sp.	−	−	−	−	nt	−	−	−	nt	−	−	−	−	−	−	−	−	−	nt	−	−	+	0.05
BT-2F1	*Bacillus cereus *	−	−	−	−	nt	−	−	−	nt	−	−	−	−	+	−	−	−	−	nt	−	−	+	0.11
MYY-2S4	*Bacillus cereus *	−	−	−	−	nt	−	−	+	nt	+	−	−	−	−	+	−	−	+	nt	−	+	+	0.32
MYY-2W7	*Bacillus* sp.	−	−	−	−	nt	−	−	+	nt	+	−	−	+	+	+	−	+	+	nt	−	−	−	0.37
MYY-2F7	*Bacillus megaterium *	−	−	−	−	nt	−	−	+	nt	+	−	−	−	−	−	−	−	+	nt	−	−	−	0.16
MYY-S10	*Bacillus megaterium *	−	−	−	−	nt	−	−	−	nt	−	−	−	−	−	−	−	−	+	nt	−	−	−	0.05
MYY-2S5	*Bacillus* sp.	−	−	−	−	nt	−	−	−	nt	−	−	−	−	−	−	−	−	−	nt	−	−	−	0.00
MYY-S9	*Bacillus cereus *	−	−	−	−	nt	−	−	+	nt	+	−	−	+	+	−	−	+	+	nt	−	−	−	0.32
MYY-S3	*Bacillus pumilus *	−	−	−	−	nt	−	−	−	nt	−	−	−	−	+	−	−	−	−	nt	−	−	−	0.05
LBG-S4	*Bacillus subtilis *	−	−	−	−	nt	−	−	−	nt	−	−	−	−	−	−	−	−	−	nt	−	−	−	0.00
LBG-2S5	*Bacillus infantis *	−	−	−	−	nt	−	−	+	nt	−	−	−	−	+	−	−	−	−	nt	−	−	−	0.11
LBG-S7	*Bacillus pumilus *	−	−	−	−	nt	−	−	−	nt	−	−	−	−	+	−	−	−	−	nt	−	−	−	0.05
LBG-2S10	*Bacillus* sp.	−	−	−	−	nt	−	−	−	nt	−	−	−	−	−	−	−	−	−	nt	−	−	−	0.00
LBG-P6	*Bacillus pumilus *	−	−	−	−	nt	+	−	+	nt	−	−	−	−	−	+	−	−	+	nt	−	+	+	0.32
LBG-W8	*Bacillus vietnamensis *	−	−	−	−	nt	−	−	−	nt	−	−	−	−	+	−	−	−	+	nt	−	+	−	0.16
LBG-2S8	*Bacillus jeotgali *	−	−	−	−	nt	−	−	−	nt	−	−	−	−	−	−	−	−	−	nt	−	−	−	0.00
SPD-P10	*Bacillus cereus *	−	−	−	−	nt	−	−	+	nt	+	−	−	+	+	+	−	−	+	nt	−	−	−	0.32
SPD-P4	*Bacillus cereus *	−	−	−	−	nt	−	−	−	nt	−	−	−	+	+	−	−	−	−	nt	−	−	+	0.16
SPD-2S3	*Bacillus cereus *	−	−	−	−	nt	−	−	−	nt	−	−	−	+	+	−	−	−	−	nt	−	−	+	0.16
SPD-2S4	*Bacillus cereus *	−	−	−	−	nt	+	−	+	nt	+	−	−	+	+	+	−	+	+	nt	−	+	+	0.53
SPD-S8	*Bacillus* sp.	−	−	−	−	nt	−	−	−	nt	−	−	−	−	−	−	−	−	−	nt	−	−	−	0.00
SPD-2W4	*Bacillus cereus *	−	−	−	−	nt	−	−	−	nt	+	−	−	−	+	−	−	−	−	nt	+	−	+	0.21
LBG-2P9	*Microbacterium* sp.	−	−	−	−	nt	−	−	−	nt	−	+	+	−	+	−	−	−	−	nt	−	+	−	0.21
SPD-S7	*Exiguobacterium profundum *	−	−	−	−	nt	−	−	−	nt	−	−	−	−	−	−	−	−	−	nt	−	−	−	0.00
BT-2W7	*Comamonas testeroni *	−	−	−	−	nt	−	−	+	nt	+	−	−	−	−	+	−	−	+	nt	−	+	+	0.32
BT-2S6	*Exiguobacterium indicum *	−	−	−	−	nt	−	−	+	nt	+	−	−	−	−	+	−	−	+	nt	−	+	+	0.32

Total isolates tested		90	90	90	71	20	85	81	51	39	74	91	80	81	90	71	91	85	61	20	75	71	51	
Total resistant isolates		5	2	1	6	17	7	5	24	15	42	12	4	25	34	30	13	8	25	6	3	26	22	
Percentage of resistant isolates (%)		5.6	2.2	1.1	8.5	85.0	8.2	6.2	47.1	38.5	56.8	13.2	5.0	30.9	37.8	42.3	16.5	9.4	41.0	30.0	4.0	36.6	43.1	

Note: isolate code BT: Batu Tujuh, BTL: Bintulu, LBG: Limbang, SPD: Sampadi, MYY: Miri, S: sediment, W: water, F: fish, and P: shrimp.

Symbol “+”: resistant, “−”: susceptible, “nt”: not tested, Ak: amikacin, Tob: tobramycin, CN: gentamicin, K: kanamycin, S: streptomycin, Te: tetracycline, DO: doxycycline, P: penicillin G, PRL: piperacillin, AMP: ampicillin, CIP: ciprofloxacin, NOR: norfloxacin, CRO: ceftriaxone, CAZ: ceftazidime, KF: cephalotin, IMP: imipenem, SXT: sulfamethoxazole/trimethoprim, RD: rifampin, NA: nalidixic acid, C: phenicols, F: nitrofurantoin, and E: erythromycin.
